# Ascitic Microbiota Composition Is Correlated with Clinical Severity in Cirrhosis with Portal Hypertension

**DOI:** 10.1371/journal.pone.0074884

**Published:** 2013-09-25

**Authors:** Geraint B. Rogers, Christopher J. van der Gast, Kenneth D. Bruce, Peter Marsh, Jane E. Collins, Julian Sutton, Mark Wright

**Affiliations:** 1 Institute of Pharmaceutical Science, King’s College London, London, United Kingdom; 2 NERC Centre for Ecology and Hydrology, Wallingford, United Kingdom; 3 Health Protection Agency, Southampton General Hospital, Southampton, United Kingdom; 4 Hepatology Group, Southampton General Hospital, Southampton, United Kingdom; Columbia University, United Kingdom

## Abstract

Identification of pathogenic bacteria in ascites correlates with poor clinical outcomes. Ascites samples are commonly reported culture-negative, even where frank infection is indicated. Culture-independent methods have previously reported bacterial DNA in ascites, however, whether this represents viable bacterial populations has not been determined. We report the first application of 16S rRNA gene pyrosequencing and quantitative PCR in conjunction with propidium monoazide sample treatment to characterise the viable bacterial composition of ascites. Twenty five cirrhotic patients undergoing paracentesis provided ascites. Samples were treated with propidium monoazide to exclude non-viable bacterial DNA. Total bacterial load was quantified by 16S rRNA Q-PCR with species identity and relative abundance determined by 16S rRNA gene pyrosequencing. Correlation of molecular microbiology data with clinical measures and diagnostic microbiology was performed. Viable bacterial signal was obtained in 84% of ascites samples, both by Q-PCR and pyrosequencing. Approximately 190,000 ribosomal pyrosequences were obtained, representing 236 species, including both gut and non gut-associated species. Substantial variation in the species detected was observed between patients. Statistically significant relationships were identified between the bacterial community similarity and clinical measures, including ascitic polymorphonuclear leukocyte count and Child-Pugh class. Viable bacteria are present in the ascites of a majority of patients with cirrhosis including those with no clinical signs of infection. Microbiota composition significantly correlates with clinical measures. Entry of bacteria into ascites is unlikely to be limited to translocation from the gut, raising fundamental questions about the processes that underlie the development of spontaneous bacterial peritonitis.

## Introduction

Bacterial infection is increasingly recognised to be a key driver in the development of complications in end stage liver disease. Spontaneous bacterial peritonitis (SBP) has been thought to result from translocation of gut bacteria into previously sterile ascites. Such infections have range of adverse outcomes, including hepatic encephalopathy and changes in portal pressure [1]. SBP occurs in up to 25% of patients with cirrhosis and ascites [2] with an in-hospital mortality rate ranging from 20 to 40% [3].

However, even where thorough microbiological practice is employed, ascitic fluid culture is reported as negative in ∼20–60% of patients with clinical manifestations suggestive of SBP, including elevated ascites polymorphonuclear leukocyte (PMN) counts (≥250 cells/mm^3^) [4], [5]. Further, patients with a PMN count ≥250 cells/mm^3^ with culture-negative ascites have similar signs, symptoms and mortality rates as patients whose ascites is culture positive [6]. This failure to routinely identify an infective agent means that empirical antibiotic treatment for SBP is initiated when objective evidence of a local inflammatory reaction is present, commonly without knowledge of the causative organisms or their antibiotic susceptibility [6], with success inferred from improvements in clinical measures, rather than through the direct observation of an impact on the pathogen. The absence of a detailed understanding of the bacteria that are present in ascites, and their role in the development of SBP, and whether a preclinical, bactero-ascitic phase is likely to precede SBP might exist, greatly hampers the development of more effective treatment regimens.

The limitations of culture-based diagnostics are increasingly well recognised [7], [8] with failure to identify a causative agent where infection is apparent being shown to occur in many clinical scenarios [9]. Such discrepancies have led to an increased deployment of culture-independent, molecular techniques, based on the detection of nucleic acid sequence signatures. The application of nucleic acid based assays to the analysis of ascites has shown that merely detecting the presence of bacterial DNA can be clinically informative, acting as a surrogate marker of bacterial translocation [10]. Further, after certain exclusions, the presence of bacterial DNA has been shown to independently predict 12-month mortality in patients with culture-negative, non-neutrocytic ascites [11].

In addition to the detection of bacterial signal, DNA-based approaches can also be used to both quantify and identify the bacterial species present. Here, approaches typically exploit the phylogenetically informative 16S ribosomal RNA gene, and include quantitative (Q) PCR (a means of determining bacterial load) and pyrosequencing (a massive parallel sequencing approach that allows the rapid, high depth characterisation of bacterial composition). Such approaches have been applied across a wide range of clinical contexts, including the analysis of intestinal microbial communities of cirrhotic patients [12], and the identification of bacterial species present in ascites [13] (although the presented study is the first reported use of pyrosequencing in this context). However, whilst having the potential to be greatly informative, such molecular techniques are weakened by their inability to distinguish between DNA present in viable bacterial cells, from that present in dead bacteria or in the extracellular environment. When applied to a clinical context, particularly where antimicrobials are being deployed, this inability is critical. A solution to this problem is to treat samples with propidium monoazide (PMA) prior to the extraction of DNA, a process that prevents the contribution of DNA other than that present in viable bacterial from contributing to subsequent PCR-based analysis. Such an approach has been shown to be effective in a range of contexts [14]–[23].

The aim of this study was to apply a molecular approach to the characterisation of the bacterial content of ascites that both excludes non-viable cells and allows the detection of bacteria that are refractory to culture *in vitro*. We hypothesised that: 1) suppression of signal derived from naked DNA or DNA present in non-viable bacteria would result in a reduction in bacterial signal compared to previous culture independent studies; 2) despite this reduction, viable bacterial cells would be detectable in a significant proportion of ascites, including those reported to be culture negative; 3) characteristics of the ascitic bacterial community composition determined in this way would be related to clinical measures of disease.

Here we present the first application of culture-independent techniques in conjunction with PMA sample treatment to characterise the bacterial composition of ascites. A combination of 16S rRNA gene pyrosequencing and 16S rRNA gene Q-PCR to identify and quantify the bacteria present, with the resulting data correlated with clinical measures of disease.

## Materials and Methods

### Clinical Samples

Ascites samples were obtained from 25 consecutive cirrhotic patients undergoing clinically indicated therapeutic or diagnostic paracentesis for ascites at Southampton General Hospital, Hampshire, UK, under full ethical approval (08/H0502/119 National Research Ethics Service Southampton & South West Hampshire Research Ethics Committee A) (Table 1). All participants provided written informed consent, using a consent procedure approved by the ethics committee.

**Table 1:** Patient information for the 25 patients enrolled in the study.

Sample	Age	Gender	Aetiology of cirrhosis	Ascitic PMN	Diagn. microbiology	Antibioticsat time of sampling	WCC	Neutrophil count	Platelets	Ascites	Child-Pugh Score	Child-Pugh Class	Creatinine	INR	Bilirubin	MELD Score
1	60	Male	ALD	200	neg	nor	4.6	3.3	72	severe	10	C	55	1.7	114	15
2	65	Female	ALD	300†	neg	cef, met	7.2	5.4	223	mild	6	A	94	1.5	24	13
3	71	Male	ALD	200	neg	taz	12	7.3	112	mod	8	B	133	1.7	187	25
4	67	Male	ALD	300†	neg	nor	7.6	6.1	183	severe	10	C	98	1.4	20	12
5*	84	Male	NASH	200	neg	none	3.5	2.5	186	mod	6	A	101	1.2	19	10
6	54	Male	ALD	200	neg	none	3.8	3.2	62	mod	6	A	189	1.2	31	18
7	56	Male	ALD	200	neg	nor	3.9	2.7	113	severe	7	B	48	1.2	10	1
8	75	Male	NASH	100	neg	none	2.4	1.7	72	mod	8	B	168	1.5	34	20
9	51	Male	ALD	100	neg	cip, cef, met	12.8	8.5	73	mild	8	B	53	1.6	50	11
10	44	Male	ALD	200	neg	none	4.7	2.8	97	mod	6	A	53	1.5	65	11
11	67	Male	ALD	100	neg	nor	6	4.7	94	severe	8	B	205	1.2	18	17
12	68	Male	ALD	200	neg	nor	3.2	1.9	73	severe	13	C	70	1.8	69	16
13*	90	Female	NASH	100	neg	none	4.9	3.3	159	severe	7	B	143	1	16	11
14	58	Male	ALD	nottested		cef, met	8	6.7	302	moderate	7	B	81	1.2	323	19
15	65	Female	Autoimmune hepatitis	1600†	neg	cip	9.9	7.4	137	mod	7	B	51	1.5	59	10
16	27	Female	Autoimmune Hepatitis	300†	neg	none	3.4	2.3	50	mod	8	B	39	not done	39	
17	61	Female	NASH	300†	neg	cip, met	5.8	4	31	severe	13	C	88	1.9	55	18
18*	37	Male	ALD	200	neg	cef, met	6.7	4.4	138	severe	10	C	65	1.6	99	15
19*	61	Female	Auto mmune hepatitis	400†	neg	nor	10.1	7.1	339	mod	9	B	65	1.4	16	7
20	43	Female	ALD	100	neg	none	9.4	7	169	severe	12	C	63	not done	449	
21	53	Male	ALD	400†	neg	cef, met	8.2	5.9	155	severe	7	B	104	1.4	33	14
22	59	Female	HCV	100	neg	cef, met	10.2	6.4	100	mild	8	B	149	2.2	22	21
23	76	male	ALD	100	neg	cef, met	13.8	10.6	154	severe	11	C	87	2	not done	
24	69	male	ALD	100	neg	cef, met	4.2	3	49	mod	5	A	59	1.3	28	7
25	47	male	ALD	300†	neg	cip	11.2	8.2	218	mod	8	B	54	1.5	410	18

Asterisks denote samples for which no sequencing data was obtained. “cef” - cefuroxime, “met” - metronidazole, “nor” - norfloxacin, “cip” - ciprofloxacin, ALD- Alcoholic liver disease. NASH- Non Alcoholic SteatoHepatitis, HCV- Hepatitis C virus.

† - denotes ascitic PMN >250 cells/mm^3^.

Child Pugh Score was determined by an experienced hepatologist (MW). Bloods were drawn on the day of the procedure. Ascites was drawn under aseptic conditions from the flank. PMN estimation and culture-based microbiology were performed as part of standard clinical practice [6]. Approximately 20 ml of ascitic fluid from each patient was centrifuged at 6,000×g for 10 min, with the resulting cell pellets resuspended in 0.5 ml of sterile phosphate-buffered saline (PBS) and stored at −80°C prior to culture-independent analysis.

### Sample Processing

Resuspended cell pellets were treated with PMA based on the method described previously [19]. Negative control samples, comprised of DNA-free water that had been used to rinse empty ascites collection vessels, were processed in parallel to clinical samples. Briefly, PMA was dissolved in 20% dimethyl sulfoxide to create a stock concentration of 20 mmol/L, 1.25 µL of which was added to 500 µL of resuspended cells. Cells were incubated for 15 min at room temperature, with occasional shaking, followed by the addition of a further 1.25 µL of PMA solution, and a further 15 min incubation. Following incubation, pellets were exposed to light for 10 min using an LED Active Blue equipment light source (Ingenia Biosystems, Terrassa, Spain). Total DNA extraction was then performed as described previously [13]. Full details of the protocol used are provided as Methods S1.

### Quantitative PCR

Total bacterial density was determined using a Taqman assay, in which a 466 bp fragment of the 16S ribosomal RNA gene was amplified. This assay was described previously [24], and applied in the analysis of ascites [13]. Full details of the protocol used are provided as Methods S1.

### 16S rRNA Gene Pyrosequencing

Bacterial tag-encoded FLX amplicon pyrosequencing (bTEFAP) was performed as described previously [22]. A full description of the methodology is provided as Methods S1.

### Statistical Analysis

Full details of statistical analysis are provided as Methods S1. A number of statistical tools used in the analysis of microbial communities to assess measures of diversity and relative abundance, as well as to assess similarity between communities in different samples, were employed here. In brief, species richness (S*) was estimated as previously described [25]. Differences in S* were computed using a re-sampling randomization method, as previously described [26]. The Sørensen presence/absence and Bray-Curtis quantitative indices of similarity and subsequent average linkage clustering of community profiles were performed using the PAST (version 2.16). Similarity of Percentages (SIMPER) analysis, used to determine the contribution of each species to the observed similarity between samples (Bray-Curtis measure), was performed as described previously [27]. Mantel tests were performed as previously described [28].

## Results

All clinical samples included in this study were reported as negative for bacterial growth based on standard diagnostic microbiological analysis. Patients 1 and 3 had previously provided samples that had reported “Gram positive cocci”.

Prior to DNA extraction, all samples were PMA treated to limit the contribution of DNA from sources other than viable bacterial cells. No bacterial signal was subsequently detected in any of the negative controls. In addition, whilst PMA treatment has been validated previously as a means of limiting amplification to live cell extracted DNA, it was further demonstrated to prevent the amplification of both Gram positive and Gram negative bacterial strains pelleted from suspension in portions of sterile ascitic fluid (Figure S1).

16S rRNA Q-PCR resulted in amplification in 21 of the 25 samples, with no amplification recorded for samples from patients 5, 13, 18 and 19. Viable bacterial densities for the 21 samples where amplification was achieved ranged from to 1.5×10^3^ to 7.2×10^5 ^cfu/ml equiv., with a mean bacterial density of 1.5×10^5 ^cfu/ml equiv (std dev 1.8×10^5^, n = 21). No significant relationship was observed between viable bacterial load and clinical measures, including ascitic fluid white cell count, blood total white cell count, blood neutrophil count, serum creatinine, survival, and time until other clinical events (SBP, as measured by the occurrence of an ascitic fluid PMN >250 cells/mm^3^; and acute gastrointestinal bleeding requiring transfusion) (Pearson’s correlation, *P*>0.05 in all instances).

**Figure 1:**
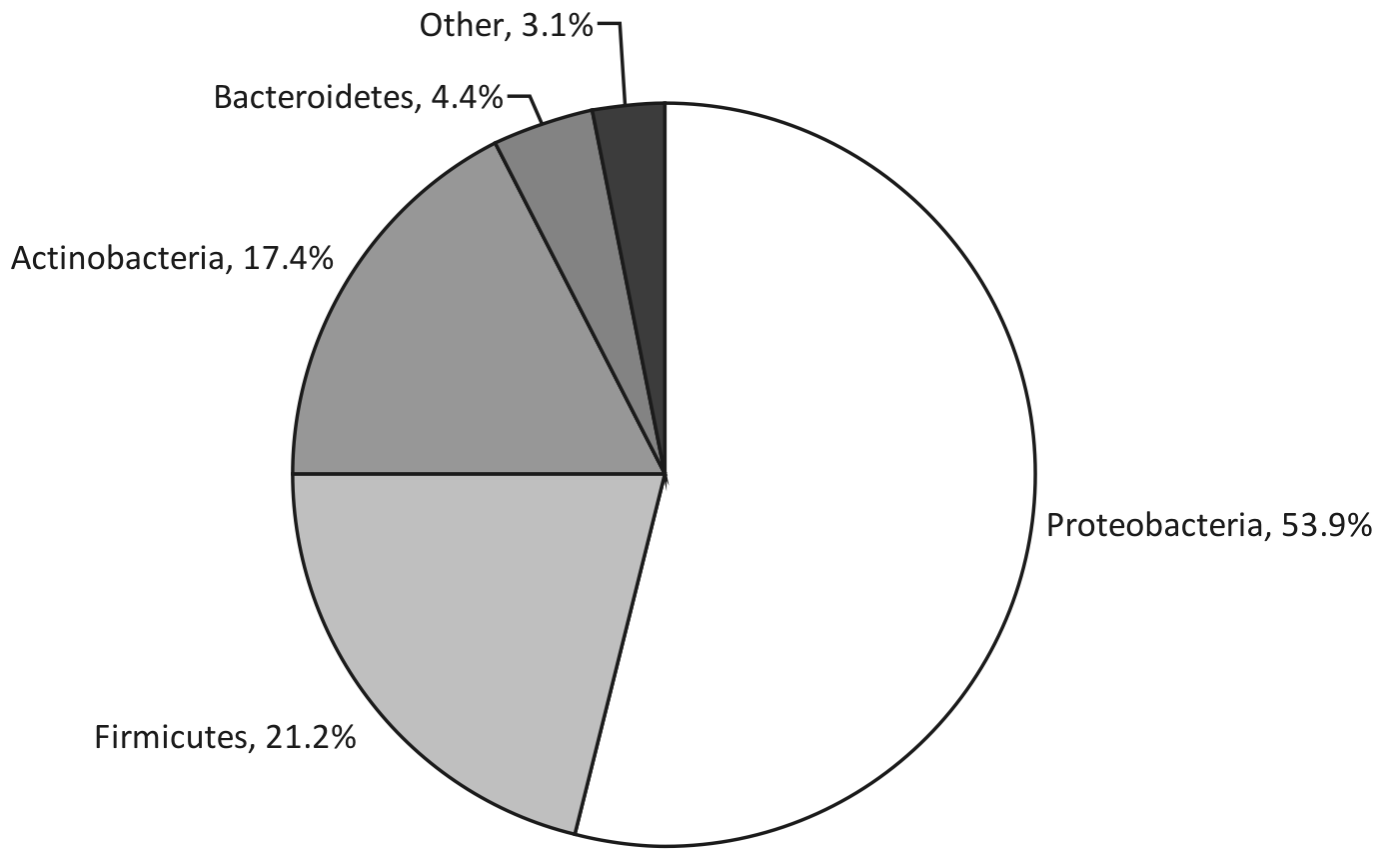
Phyla represented by 16S rRNA gene sequences obtained.

DNA extracts for all 25 samples were next subjected to 16S rRNA gene pyrosequencing. In keeping with the results of Q-PCR analysis, PCR amplification, and subsequent pyrosequencing, were successful for 21 of the 25 samples. A total of 189,960 16S rRNA sequences were obtained, with an average of 9045 sequences per sample (SD ±8642, *n* = 21, range 1901–39522). Members of the phyla Proteobacteria dominated the sequences obtained (53.9%), followed by Firmicutes (21.2%), Actinobacteria (17.4%), and Bacteroidetes (4.4%), with other phyla representing 3.1% of sequences (Figure 1). In total, 236 unique bacterial species were detected across the 21 samples analysed. Species identities, occupancy (the number of samples a given species was detected in) and anaerobicity are shown in Table S1.

**Figure 2:**
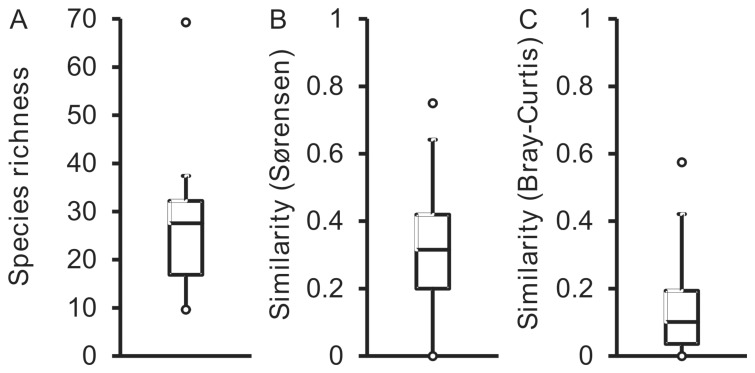
Box plot comparisons of bacterial diversity, bacterial community membership, and bacterial community structure for the 21 cross-sectional samples (A, B, and C, respectively). Richness was calculated with a uniform re-sample size of 1901 sequences following 1000 iterations. Community membership and structure were calculated using the Sørensen (presence/absence) and Bray-Curtis (quantitative) indices of similarity, respectively. The top and bottom boundaries of each box plot indicate the 75^th^ and 25^th^ quartile values, respectively, and lines within each box represent the 50^th^ quartile (media) values. Ends of whiskers represent interquartile range and open circles mark the lowest and highest values in each instance.

**Figure 3:**
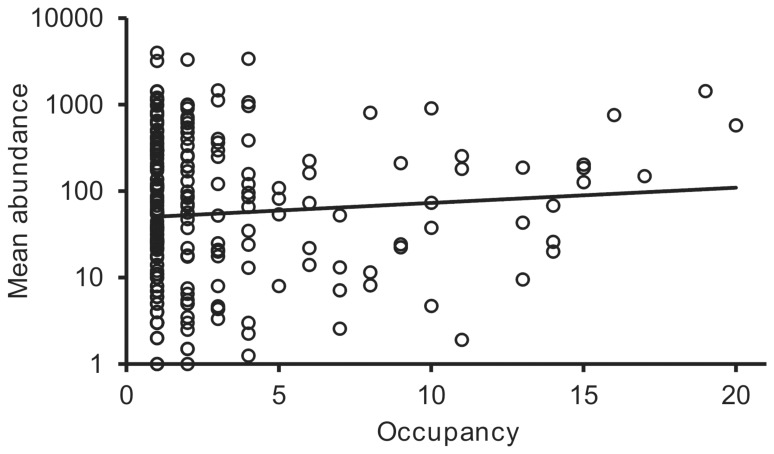
Distribution of bacterial species across patients. Given is occupancy, (the number of samples for which each bacterial species was observed), plotted against mean species abundance (log_10_ scale) across all 21 samples (*r* ^2^ = 0.084, *F*_1,234_ = 1.38, *P* = 0.242).

Bacterial species richness (number of species) was calculated based on uniform re-sample of 16S rRNA gene sequences, and ranged from 10 to 69 species per sample with a mean richness of 26.7 (SD ±13.0, *n = *21) (Figure 2). To investigate whether the frequent detection of bacterial species within the patient group (expressed as occupancy) was positively correlated to their relative abundance within the samples, the distribution of bacterial species across the patient group was determined. Here, occupancy was found to not have a significant relationship with mean species abundance (*r* ^2^ = 0.084, *F*_1,234_ = 1.38, *P* = 0.242) (Figure 3). Further, a high number of taxa that were detected with high relative abundances were unique to a given individual patients.

Similarities and differences in bacterial community membership and structure between patient samples were assessed using the Sørensen (based on the presence/absence of species) and Bray-Curtis (based on presence/absence of species and their relative abundance) indices of similarity, respectively (Figure 2). The mean similarity of bacterial communities taken pairwise using the Sørensen index was 0.31 with a standard deviation of ±0.14 (n = 210 pairwise comparisons). For the Bray-Curtis index, the mean similarity was 0.14±0.13 (Figure 2). Clustering of patient community profiles using both measures are shown as cluster diagrams in Figure S2. Both box plots and cluster analyses revealed low levels of similarity in terms of community membership and structure between patients.

SIMPER analysis of bacterial community similarity (Bray-Curtis index) between patients was performed to determine the average contribution of a given species to the average similarity between the samples analysed. Whilst overall levels of similarity between the bacterial profiles from different patients were low, species that contributed most to the similarity observed are listed in Table 2. Here, species occupancy, the mean percentage abundance of species across the samples in which a species was detected, the percentage contribution (mean contribution divided by mean similarity across samples) and percentage cumulative contribution, are shown in for the five species with the highest percentage contribution to overall similarity (*Acidovorax facilis, Stenotrophomonas maltophilia, Propionibacterium acnes, Streptococcus pneumoniae,* and *Ralstonia pickettii*).

**Table 2:** Similarity of Percentages (SIMPER) analysis of bacterial community similarity (Bray-Curtis index) between patients.

Species	Occupancy	% Mean Abund.	Mean Cont.	% Cont.	Cum. %
*Acidovorax facilis*	19	15.53	5.79	42.50	42.50
*Stenotrophomonas maltophilia*	16	6.64	2.19	16.09	58.60
*Propionibacterium acnes*	20	4.29	1.46	10.75	69.35
*Streptococcus pneumoniae*	10	4.93	0.84	6.19	75.54
*Ralstonia pickettii*	15	2.57	0.59	4.35	79.90

Given is occupancy, or number of patients a given species was detected in. Mean % abundance of species across the samples it was observed to occupy. Mean contribution represents the average contribution of a given species to the average similarity between samples (overall mean = 13.6%). Percentage contribution is the mean contribution divided by mean similarity across samples. The list of species is not exhaustive so cumulative % value does not sum to 100%. An arbitrary threshold of a mean contribution of 0.5 was used as a cut-off.

Correlation of bacterial community similarity (measured using Sørensen or Bray-Curtis indices of similarity) with a number of clinical measures (antibiotic therapy, Child-Pugh Score, and PMN count) was performed using Mantel tests. The Mantel statistic (*r*) and the significance of differences between Mantel regression coefficients and zero following 9,999 permutations, *P*, are shown in Table 3. Statistically-significant relationships were identified between Bray-Curtis similarity indices and ascitic PMN (*P*<0.0001), SBP diagnosis (*P = *0.002), Child-Pugh class (*P = *0.011), When Sørensen similarity indices were used, a significant relationship was identified with Child-Pugh class (*P = *0.003). Partial Mantel tests indicated significant relationships between Bray-Curtis similarity indices and both ascitic PMN (*P*<0.0001) and Child-Pugh class (*P = *0.003) when one of these two factors was controlled for. No correlation was observed between bacterial community similarity and whether subjects had alcoholic liver disease. In summary, the ascitic microbiology is similar amongst patients with less severe disease, as is the ascitic microbiology amongst patients with more severe disease.

**Table 3:** Summary statistics for Mantel and partial Mantel tests between community similarity and clinical factors.

Test	Similarity(*A*)	Parameter(*B*)	Control for(*C*)	*r*	*P*
Mantel	Sørensen	Ascitic PMN		0.086	0.207
		SBP		−0.096	0.078
		Child-Pughclass		−0.189	0.003*
Mantel	Bray-Curtis	Ascitic PMN		−0.230	<0.0001*
		SBP		−0.193	0.002*
		Child-Pughclass		−0.155	0.011*
partialMantel	Bray-Curtis	Ascitic PMN	Child-Pughclass	−0.253	<0.0001*
		Child-Pughclass	Ascitic PMN	−0.190	0.003*

The Mantel statistic *r*(*AB*) estimates the correlations between two proximity matrices, *A* and *B*. Whereas, the partial mantel statistic *r*(*AB*.*C*) estimates the correlation between *A* and *B* whilst controlling for the effects of *C*. Given are bacterial community similarity (Sørensen and Bray-Curtis indices of similarity) and also differences in clinical factors. Also given is *P* to ascertain whether the Mantel regression coefficients were significantly different from zero following 9,999 permutations. Asterisks denote those relationships that were significant at the *P*<0.05 level.

## Discussion

This is the first culture independent survey of the ascitic microbiota using the 16S rRNA gene pyrosequencing. Distinct viable bacterial community profiles were found in the majority of the patients studied. Differences in structure and membership of these communities correlated with disease severity and ascitic PMN.

High rates of culture negativity in the diagnostic microbiological analysis of ascites samples, even where there are clinical signs of infection, suggest that such methods are poor in characterising the bacteria present. Further support for this was provided by previous culture-independent analysis of ascites samples which reported the presence of DNA derived from a diverse array of bacterial species [13]. However, in the absence of a strong correlation between the presence of bacterial DNA and the occurrence of SBP, we hypothesised that naked DNA present in ascitic fluid, or DNA present in non-viable bacteria, may contribute significantly to such PCR-based signals, potentially obscuring any relationship between microbes and clinical parameters. To limit culture-independent analysis to viable bacterial cells, PMA sample treatment was employed. Prior to its application to ascites samples, we first confirmed the efficacy of this approach by spiking sterile ascitic fluid with heat-killed Gram negative and Gram positive bacteria at levels comparable to those reported previously in this context. PCR amplification of bacterial DNA extracted from these samples was found to be prevented.

Further evidence of the contribution of DNA from non-viable sources to the culture-independent analysis of ascites comes from comparison of the data presented here with that from an earlier study [13] where a comparable sample set was analysed but without PMA pre-treatment. The mean bacterial load reported in this previous study was found to be significantly higher than those reported here, suggesting DNA other than that present in viable bacteria had made a substantial contribution to analysis. This finding also provides a potential explanation for the previous report of only partial correlation between the presence of bacterial DNA in ascites and prognosis [29]. Whilst no approach can completely exclude nucleic acids from non-viable bacteria to PCR-based analysis, the approach applied here substantially improves it.

As hypothesised, considerable diversity was observed by 16S rRNA gene 454 pyrosequencing, despite the reduction in amplifiable DNA that resulted from PMA treatment. More than 230 separate bacterial species detected in the sample set. Whilst the bacterial loads determined by Q-PCR were relatively low, the presence of viable bacteria within ascites, in the absence of clinical SBP, suggests that the presence of bacteria is necessary but not sufficient for this condition to develop. Therefore, all of the factors required for the onset of SBP remain to be identified.

The presence of such a diverse array of bacterial species raises the question of their origin. The principle source is believed to be the gut, via translocation from the gastrointestinal lumen to the mesenteric lymph nodes, and the subsequent systemic circulation and seeding into ascites [29]. However, in addition, it may be possible for bacteria to translocate from other areas of the body where bacterial populations overlay endothelia that may have become porous to bacterial movement or be introduced via indwelling devices. To some extent, the relative contribution of these different potential routes can be inferred from the types of bacterial species detected. Overall, 97% of the species identified here were from within one of the four key phyla that comprise the human microbiota; Proteobacteria, Firmicutes, Actinobacteria, and Bacteroidetes. However, amongst these, Proteobacteria were dominant, representing more than 60% of sequences detected. The gut microbiota in healthy individuals is dominated by Bacteroidetes and Firmicutes [30]. However, in patients with liver cirrhosis, there is a decrease in the relative abundance of Bacteroidetes and an increase in the relative abundance of Proteobacteria and Fusobacteria [12], [31]. Therefore, to some extent, such a shift in gut microbiota composition could account for the phylum level distribution of the bacterial species reported here.

Examined in more detail, the increase in the relative abundance of Proteobacteria in the intestinal microbiota of cirrhotic patients is primarily the result of increases in Enterobacteriaceae and Pasteurellaceae. In addition, there are increases in Streptococcaceae and Veillonellaceae [12], with Streptococcaceae in particular shown to have a positive correlation trend with cirrhosis severity [12]. Species belonging to Streptococcaceae were particularly widely detected in the samples analysed here, in addition to other species common within the intestinal microbiota, such as members of the genera *Bacteroides, Bifidobacterium, Clostridium, Eubacterium, Fusobacterium,* and *Ruminococcus*, supporting such a gut translocation model.

However, in addition to gut-associated microbes, many other species were detected that are far less likely to be present as the result of translocation from the intestine, even where it is occupied by a modified microbiota. For example, members of a number of aerobic genera more commonly associated with opportunistic infections, including *Acinetobacter*, *Pseudomonas*, *Ralstonia*, *Stenotrophomonas*, and *Staphylococcus,* were detected. In other cases, members of genera recognised as commonly colonising the skin, such as members of the genus *Propionibacterium,* were present. Therefore, whilst translocation of bacteria from the gut may be an important route by which bacteria can enter ascites, the data presented here suggest that it is not the only one.

A low level of microbiota similarity was observed between patients. Similarity was also found to be low when the relative abundance of these species was taken into account. This finding suggests an element of stochasticity in the bacterial species present in ascitic fluid. If so, this has by extension important implications for treatment in that one antibiotic regime is unlikely to “cover” adequately such a diverse range of species. In the most part, these species were classed as opportunist pathogens, and as such, these would not be uncommon in a nosocomial environment.

Despite the divergence in bacterial species present in different patients, microbiota membership and structure correlated closely with differences in ascitic PMN and Child-Pugh class. Microbiota similarity between patients, as measured by the Sorensen index (species presence/absence) also correlated with these two clinical factors (Table 3). No other clinical factors significantly correlated with either measure of microbiota similarity. Partial Mantel tests revealed that both differences in ascitic PMN and Child-Pugh class both significantly correlated with microbiota similarity (when species relative abundance was taken into account) even when each factor was tested whilst controlling for the effects of the other. This finding suggests that there may be a relationship between the composition of the bacterial component of ascites and a patients long-term prognosis, either through the existence of factors that promote the translocation of these species to the peritoneal cavity, or by predisposing patients to SBP or disease progression. As such, this finding clearly warrants further investigation.

In detecting bacterial signal in the majority of ascitic fluid analysed, the present study is in contrast with a number of previous studies that have reported lower levels of sample positivity for bacterial DNA. Negative controls were included in each step of the analytical process, including PMA treatment, DNA extraction, and PCR amplification, with no signal detected, supporting these findings not being artefactual. Moreover, the dissimilarity of species detected as present in the patients studied was not consistent with any systematic contamination. Patient selection may be one reason why the levels of sample positivity for bacterial DNA detected here differs from some other studies. Some studies have excluded patients with ascitic PMN counts >250/µl [10], [11], whereas here, no attempt was made to stratify patients, with those included representing a cross-section of the patient population. In many cases, these patients had indications of frank infection. In addition, with the increasing sophistication of the molecular detection systems, it is likely that detection thresholds of the Q-PCR approach used here is lower than that previously achievable. Where no bacterial signal was obtained from samples analysed in the current study, a number of factors could contribute to the failure of PCR amplification. For example, of the four patients who provided PCR-negative samples three had non-alcoholic liver disease. Here, the relative absence of intestinal permeability and bacterial translocation [32] may have resulted in lower levels of bacteria in ascites. The fourth PCR-negative patient was receiving broad spectrum antibiotics at the time of sampling, again, potentially reducing viable bacterial signal.

The study had a number of limitations that should be considered. Analysis was performed on samples from a relatively small group of subjects (n = 25), and application of the methodology described here to a larger patient populations would be likely to further define the relationships between microbiological and clinical measures reported. The majority of subjects were receiving some form of antibiotic therapy at the time of sampling, a factor that is likely to have influenced both the viable bacterial composition of the ascites analysed, and the likelihood of samples being culture-negative. Here, the subjects’ antibiotic treatment profile reflects the fact that they were a cross-section of the patient population. Analysis of samples obtained prior to, as well as during antibiotic therapy would help to define the impact of such therapy on the viable bacterial community in ascites. Such longitudinal analysis would also provide greater insight into the relationship between bacteriological factors and clinical progression. Finally, whilst the identities of bacteria detected suggested potential routes of entry into the peritoneal cavity, parallel analysis of stool samples, samples from skin surrounding the drain site, and from other areas from which bacteria might be derived, would provide further clarification.

Taken together, the data presented here suggest that ascitic fluid may be exposed to bacterial species from a range of sources, and that access to the peritoneal cavity is non-limited to translocation of bacteria from the gut. Further, they indicate that, whilst the presence of bacteria in ascites is not sufficient for the occurrence of SBP, a relationship exists between the composition of the bacterial species content of ascites and clinical measures of disease. Investigations of the changes that occur in these bacterial communities during the development of SBP are now clearly warranted.

## Supporting Information

Figure S1 **Validation of PMA treatment in ascites fluid.** Lane 1– no template negative control. Lanes 2, 4, and 6– amplification reactions using DNA extracted from heat-killed *A. baumanii*, *S. aureus*, and *E. coli*, respectively, as template. Lanes 3, 5, and 7 - amplification reactions using DNA extracted from PMA treated heat-killed *A. baumanii*, *S. aureus*, and *E. coli*, respectively.
(DOCX)

Figure S2 **Cluster diagrams of bacterial community composition in the 21 patients.** Patient species profiles were compared using the (A) Sørensen and (B) Bray-Curtis quantitative indices of similarity and average linkage clustering.
(TIF)

Table S1 **Bacterial species sampled across the 21 cross-sectional samples.** Occupancy denotes the number of samples a given species was detected in. Respiration - Ae, denotes aerobe; An, Anerobe^1^.
(DOCX)

Methods S1 **Full details of methods used in nucleic acid extraction, quantitative PCR, 16S rRNA gene pyrosequencing, and statistical analysis.**
(DOC)
