# Supplementary material for: Ascitic Microbiota Composition Is Correlated with Clinical Severity in Cirrhosis with Portal Hypertension
Source: PLoS One. 2013 Sep 25;8(9):e74884. doi: 10.1371/journal.pone.0074884 (PMC3783492; doi:10.1371/journal.pone.0074884)
Supplement: Figure S1 — Validation of PMA treatment in ascites fluid. Lane 1– no template negative control. Lanes 2, 4, and 6– amplification reactions using DNA extracted from heat-killed A. baumanii, S. aureus, and E. coli, respectively, as template. Lanes 3, 5, and 7 - amplification reactions using DNA extracted from PMA treated heat-killed A. baumanii, S. aureus, and E. coli, respectively. (DOCX) [file pone.0074884.s001.docx]

| **Class** | **Order** | **Family** | **Taxon name** | **Occup.** | **Resp.** |
| --- | --- | --- | --- | --- | --- |
| Acidobacteria | Acidobacteriales | Acidobacteriaceae | *Terriglobus roseus* | 2 | Ae |
| Actinobacteria | Actinomycetales | Actinomycetaceae | *Actinomyces hongkongensis* | 2 | Ae |
|  |  |  | *Actinomyces johnsonii* | 1 | Ae |
|  |  |  | *Actinomyces oris* | 1 | Ae |
|  |  |  | *Actinotalea fermentans* | 1 | Ae |
|  |  | Bifidobacteriaceae | *Bifidobacterium longum* | 2 | An |
|  |  |  | *Bifidobacterium thermacidophilum* | 1 | An |
|  |  | Brevibacteriaceae | *Brevibacterium aureum* | 1 | Ae |
|  |  | Corynebacteriaceae | *Corynebacterium afermentans* | 3 | Ae |
|  |  |  | *Corynebacterium amycolatum* | 1 | Ae |
|  |  |  | *Corynebacterium confusum* | 1 | Ae |
|  |  |  | *Corynebacterium genitalium* | 2 | Ae |
|  |  |  | *Corynebacterium kroppenstedtii* | 1 | Ae |
|  |  |  | *Corynebacterium mucifaciens* | 3 | Ae |
|  |  |  | *Corynebacterium tuberculostearicum* | 4 | Ae |
|  |  |  | *Corynebacterium tuscaniae* | 1 | Ae |
|  |  |  | *Corynebacterium vitaeruminis* | 2 | Ae |
|  |  | Cryptosporangiaceae | *Cryptosporangium japonicum* | 1 | Ae |
|  |  | Geodermatophilaceae | *Blastococcus saxobsidens* | 4 | Ae |
|  |  | Kineosporiaceae | *Kineococcus radiotolerans* | 1 | Ae |
|  |  |  | *Kineosporia mikuniensis* | 1 | Ae |
|  |  | Microbacteriaceae | *Agrococcus jejuensis* | 2 | Ae |
|  |  |  | *Microbacterium barkeri* | 1 | Ae |
|  |  |  | *Microbacterium flavescens* | 1 | Ae |
|  |  |  | *Rathayibacter tritici* | 1 | Ae |
|  |  | Micrococcaceae | *Arthrobacter cumminsii* | 1 | Ae |
|  |  |  | *Arthrobacter nicotinovorans* | 2 | Ae |
|  |  |  | *Kocuria palustris* | 1 | Ae |
|  |  |  | *Micrococcus luteus* | 2 | Ae |
|  |  |  | *Rothia mucilaginosa* | 2 | Ae |
|  |  | Micromonosporaceae | *Micromonospora echinospora* | 1 | Ae |
|  |  |  | *Micromonospora fulviviridis* | 1 | Ae |
|  |  |  | *Virgisporangium ochraceum* | 1 | Ae |
|  |  | Nocardiaceae | *Rhodococcus rhodochrous* | 1 | Ae |
|  |  | Nocardioidaceae | *Aeromicrobium ponti* | 1 | Ae |
|  |  |  | *Marmoricola* | 1 | Ae |
|  |  |  | *Nocardioides oleivorans* | 2 | Ae |
|  |  |  | *Propionibacterium acidifaciens* | 1 | Ae |
|  |  |  | *Propionibacterium acnes* | 20 | Ae |
|  |  |  | *Propionibacterium propionicum* | 2 | Ae |
|  |  | Nocardiopsaceae | *Nocardiopsis composta* | 1 | Ae |
|  |  | Pseudonocardiaceae | *Amycolatopsis halotolerans* | 1 | Ae |
|  |  |  | *Goodfellowiella coeruleoviolacea* | 1 | Ae |
|  |  |  | *Pseudonocardia callicarpae* | 1 | Ae |
|  |  | Sanguibacteraceae | *Sanguibacter inulinus* | 1 | Ae |
|  |  | Streptomycetaceae | *Streptomyces bluensis* | 1 | Ae |
|  |  |  | *Streptomyces laceyi* | 1 | Ae |
|  |  |  | *Streptomyces thermolineatus* | 1 | Ae |
| Actinobacteria | Rubrobacterales | Rubrobacteraceae | *Rubrobacter radiotolerans* | 1 | Ae |
|  |  |  | *Rubrobacter xylanophilus* | 1 | Ae |
| Bacteroidia | Bacteroidales | Bacteroidaceae | *Bacteroides acidifaciens* | 4 | An |
|  |  |  | *Bacteroides plebeius* | 3 | An |
|  |  |  | *Bacteroides thetaiotaomicron* | 2 | An |
|  |  | Porphyromonadaceae | *Porphyromonas catoniae* | 2 | An |
|  |  |  | *Prevotella enoeca* | 1 | An |
| Flavobacteria | Flavobacteriales | Flavobacteriaceae | *Chryseobacterium daecheongense* | 8 | Ae |
|  |  |  | *Chryseobacterium hominis* | 5 | Ae |
|  |  |  | *Chryseobacterium joostei* | 15 | Ae |
|  |  |  | *Coenonia anatina* | 1 | Ae |
|  |  |  | *Flavobacterium aquatile* | 1 | Ae |
|  |  |  | *Flavobacterium columnare* | 1 | Ae |
|  |  |  | *Flavobacterium succinicans* | 2 | Ae |
|  |  |  | *Maribacter goseongensis* | 1 | Ae |
|  |  |  | *Riemerella anatipestifer* | 15 | Ae |
|  |  |  | *Sejongia* | 1 | Ae |
|  |  |  | *Tenacibaculum japonica* | 1 | Ae |
|  |  |  | *Zhouia amylolytica* | 1 | Ae |
| Sphingobacteria | Sphingobacteriales | Chitinophagaceae | *Chitinophaga arvensicola* | 2 | Ae |
|  |  |  | *Chitinophaga filiformis* | 2 | Ae |
|  |  |  | *Chitinophaga sancti* | 1 | Ae |
|  |  |  | *Segetibacter* | 1 | Ae |
| Sphingobacteria | Sphingobacteriales | Sphingobacteriaceae | *Pedobacter cryoconitis* | 1 | Ae |
|  |  |  | *Pedobacter rhizospharae* | 1 | Ae |
|  |  |  | *Sphingobacterium composta* | 1 | Ae |
| Cyanobacteria | Prochlorophytes | Prochlorococcaceae | *Prochlorococcus* | 1 | Ae |
|  |  |  | *Prochlorococcus marinus* | 1 | Ae |
| Oscillatoriophycideae | Oscillatoriales | Phormidiaceae | *Geitlerinema* | 4 | Ae |
| Bacilli | Bacillales | Alicyclobacillaceae | *Alicyclobacillus pomorum* | 1 | Ae |
|  |  | Bacillaceae | *Bacillus arbutinivorans* | 1 | Ae |
|  |  |  | *Bacillus clausii* | 1 | Ae |
|  |  |  | *Geobacillus stearothermophilus* | 4 | Ae |
|  |  |  | *Geobacillus thermoparaffinivorans* | 2 | Ae |
|  |  | Listeriaceae | *Brochothrix thermosphacta* | 3 | Ae |
|  |  | Staphylococcaceae | *Staphylococcus epidermidis* | 8 | Ae |
|  |  |  | *Staphylococcus hominis* | 2 | Ae |
|  |  | Thermoactinomycetaceae | *Thermoactinomyces vulgaris* | 1 | Ae |
|  | Gemellales | Gemellaceae | *Gemella haemolysans* | 1 | Ae |
|  |  |  | *Gemella sanguinis* | 1 | Ae |
|  | Lactobacillales | Aerococcaceae | *Abiotrophia defectiva* | 2 | Ae |
|  |  | Carnobacteriaceae | *Alloiococcus otitis* | 1 | Ae |
|  |  |  | *Carnobacterium maltaromaticum* | 3 | Ae |
|  |  |  | *Granulicatella adiacens* | 1 | Ae |
|  |  |  | *Granulicatella elegans* | 1 | Ae |
|  |  |  | *Marinilactibacillus psychrotolerans* | 1 | Ae |
|  |  | Enterococcaceae | *Enterococcus cecorum* | 1 | Ae |
|  |  | Lactobacillaceae | *Aerococcus viridans* | 1 | Ae |
|  |  | Leuconostocaceae | *Leuconostoc citreum* | 2 | Ae |
|  |  | Streptococcaceae | *Streptococcus cristatus* | 8 | Ae |
|  |  |  | *Streptococcus mitis* | 10 | Ae |
| *Table S1 continued* |  |  |  |  |  |
| **Class** | **Order** | **Family** | **Taxon name** | **Occup.** | **Resp.** |
|  |  |  | *Streptococcus oralis* | 10 | Ae |
|  |  |  | *Streptococcus pneumoniae* | 10 | Ae |
|  |  |  | *Streptococcus thermophilus* | 1 | Ae |
| Clostridia | Clostridiales | Clostridiaceae | *Anaerococcus hydrogenalis* | 1 | An |
|  |  |  | *Anaerococcus octavius* | 1 | An |
|  |  |  | *Anaerococcus prevotii* | 1 | An |
|  |  |  | *Clostridium bolteae* | 1 | An |
|  |  |  | *Clostridium botulinum* | 1 | An |
|  |  |  | *Clostridium cellobioparum* | 1 | An |
|  |  |  | *Clostridium hveragerdense* | 1 | An |
|  |  |  | *Clostridium kluyveri* | 11 | An |
|  |  |  | *Clostridium straminisolvens* | 2 | An |
|  |  |  | *Finegoldia magna* | 4 | An |
|  |  | Eubacteriaceae | *Acetobacterium carbinolicum* | 3 | An |
|  |  |  | *Eubacterium minutum* | 1 | An |
|  |  |  | *Eubacterium rectale* | 6 | An |
|  |  |  | *Eubacterium sulci* | 1 | An |
|  |  | Heliobacteriaceae | *Heliobacterium modesticaldum* | 1 | An |
|  |  | Lachnospiraceae | *Roseburia faecis* | 2 | An |
|  |  | Ruminococcaceae | *Anaerotruncus colihominis* | 1 | An |
|  |  |  | *Faecalibacterium prausnitzii* | 7 | An |
|  |  |  | *Ruminococcus torques* | 1 | An |
| Erysipelotrichi | Erysipelotrichales | Erysipelotrichaceae | *Allobaculum stercoricanis* | 1 | An |
| Negativicutes | Selenomonadales | Acidaminococcaceae | *Phascolarctobacterium* | 2 | An |
|  |  | Veillonellaceae | *Anaerospora hongkongensis* | 3 | An |
|  |  |  | *Desulfosporomusa polytropa* | 7 | An |
|  |  |  | *Dialister micraerophilus* | 1 | An |
|  |  |  | *Sporomusa ovata* | 1 | An |
|  |  |  | *Veillonella montpellierensis* | 1 | An |
|  |  |  | *Veillonella ratti* | 3 | An |
| Fusobacteria | Fusobacteriales | Fusobacteriaceae | *Fusobacterium nucleatum* | 1 | An |
| Nitrospira | Nitrospirales | Nitrospiraceae | *Nitrospira moscoviensis* | 1 | Ae |
| Planctomycea | Gemmatales | Gemmataceae | *Gemmata obscuriglobus* | 4 | Ae |
| Alphaproteobacteria | Caulobacterales | Caulobacteraceae | *Caulobacter leidyia* | 1 | Ae |
|  |  |  | *Asticcacaulis biprosthecium* | 1 | Ae |
|  |  |  | *Brevundimonas diminuta* | 1 | Ae |
|  | Rhizobiales | Aurantimonadaceae | *Aurantimonas altamirensis* | 1 | Ae |
|  |  | Bradyrhizobiaceae | *Bradyrhizobium elkanii* | 3 | Ae |
|  |  | Brucellaceae | *Ochrobactrum intermedium* | 1 | Ae |
|  |  | Hyphomicrobiaceae | *Devosia ginsengisoli* | 2 | Ae |
|  |  |  | *Devosia riboflavina* | 2 | Ae |
|  |  |  | *Hyphomicrobium* | 2 | Ae |
|  |  |  | *Rhodoplanes* | 3 | Ae |
|  |  | Methylobacteriaceae | *Methylobacterium jeotgali* | 4 | Ae |
|  |  |  | *Methylobacterium variabile* | 1 | Ae |
|  |  | Methylocystaceae | *Methylosinus trichosporium* | 2 | Ae |
|  |  | Phyllobacteriaceae | *Phyllobacterium myrsinacearum* | 2 | Ae |
|  |  | Rhizobiaceae | *Rhizobium sullae* | 1 | Ae |
|  | Rhodobacterales | Rhodobacteraceae | *Paracoccus aminophilus* | 1 | Ae |
|  |  |  | *Paracoccus versutus* | 1 | Ae |
|  | Rhodospirillales | Acetobacteraceae | *Roseomonas lacus* | 1 | Ae |
|  |  |  | *Roseomonas mucosa* | 1 | Ae |
|  |  |  | *Roseomonas terrae* | 1 | Ae |
|  | Rickettsiales | Anaplasmataceae | *Wolbachia pipientis* | 1 | Ae |
|  | Sphingomonadales | Erythrobacteraceae | *Porphyrobacter tepidarius* | 1 | Ae |
|  |  |  | *Kaistobacter terrae* | 2 | Ae |
|  |  |  | *Novosphingobium* | 11 | Ae |
|  |  |  | *Sphingobium fuliginis* | 3 | Ae |
|  |  |  | *Sphingobium yanoikuyae* | 6 | Ae |
|  |  |  | *Sphingomonas aerolata* | 1 | Ae |
|  |  |  | *Sphingomonas melonis* | 1 | Ae |
|  |  |  | *Sphingomonas yabuuchiae* | 1 | Ae |
| Betaproteobacteria | Burkholderiales | Alcaligenaceae | *Achromobacter xylosoxidans* | 3 | Ae |
|  |  | Burkholderiaceae | *Cupriavidus campinensis* | 1 | Ae |
|  |  |  | *Cupriavidus necator* | 3 | Ae |
|  |  |  | *Ralstonia pickettii* | 15 | Ae |
|  |  |  | *Roseateles depolymerans* | 7 | Ae |
|  |  | Comamonadaceae | *Acidovorax avenae* | 2 | Ae |
|  |  |  | *Acidovorax citrulli* | 4 | Ae |
|  |  |  | *Acidovorax delafieldii* | 2 | Ae |
|  |  |  | *Acidovorax facilis* | 19 | Ae |
|  |  |  | *Acidovorax temperans* | 14 | Ae |
|  |  |  | *Comamonas aquatica* | 2 | Ae |
|  |  |  | *Comamonas denitrificans* | 6 | Ae |
|  |  |  | *Comamonas testosteroni* | 13 | Ae |
|  |  |  | *Delftia* | 5 | Ae |
|  |  |  | *Hydrogenophaga palleronii* | 1 | Ae |
|  |  |  | *Pelomonas* | 2 | Ae |
|  |  |  | *Pelomonas puraquae* | 3 | Ae |
|  |  |  | *Schlegelella thermodepolymerans* | 2 | Ae |
|  |  |  | *Variovorax paradoxus* | 1 | Ae |
|  |  | Oxalobacteraceae | *Massilia timonae* | 4 | Ae |
|  | Methylophilales | Methylophilaceae | *Methylobacillus aminovorus* | 1 | Ae |
|  |  |  | *Methylobacillus flagellatus* | 4 | Ae |
|  | Neisseriales | Neisseriaceae | *Neisseria sicca* | 2 | Ae |
|  |  |  | *Neisseria subflava* | 4 | Ae |
|  | Rhodocyclales | Rhodocyclaceae | *Azonexus fungiphilus* | 1 | Ae |
|  |  |  | *Zoogloea ramigera* | 1 | Ae |
| Deltaproteobacteria | Desulfobacterales | Desulfobacteraceae | *Desulfatibacillum alkenivorans* | 1 | Ae |
|  | Desulfobacterales | Desulfovibrionaceae | *Desulfovibrio burkinensis* | 1 | Ae |
|  |  |  | *Desulfovibrio desulfuricans* | 4 | Ae |
|  | Myxococcales | Nannocystaceae | *Nannocystis exedens* | 1 | Ae |
|  |  | Polyangiaceae | *Chondromyces crocatus* | 1 | Ae |
| Gammaproteobacteria | Aeromonadales | Aeromonadaceae | *Aeromonas hydrophila* | 1 | Ae |
|  |  |  | *Aeromonas punctata* | 10 | Ae |
|  |  |  | *Aeromonas salmonicida* | 1 | Ae |
|  |  |  | *Aeromonas veronii* | 2 | Ae |
|  | Alteromonadales | Shewanellaceae | *Shewanella putrefaciens* | 4 | Ae |
|  | Chromatiales | Chromatiaceae | *Rheinheimera aquimaris* | 1 | Ae |
|  |  | Ectothiorhodospiraceae | *Thioalkalivibrio denitrificans* | 2 | Ae |
|  | Enterobacteriales | Enterobacteriaceae | *Aquamonas fontana* | 1 | Ae |
|  |  |  | *Citrobacter farmeri* | 1 | Ae |
|  |  |  | *Erwinia persicina* | 1 | Ae |
|  |  |  | *Escherichia coli* | 4 | Ae |
|  |  |  | *Klebsiella oxytoca* | 5 | Ae |
|  |  |  | *Proteus mirabilis* | 1 | Ae |
|  |  |  | *Yersinia mollaretii* | 2 | Ae |
|  | Methylococcales | Crenotrichaceae | *Crenothrix polyspora* | 1 | Ae |
|  | Pasteurellales | Pasteurellaceae | *Aggregatibacter segnis* | 1 | Ae |
|  |  |  | *Haemophilus parainfluenzae* | 1 | Ae |
|  | Pseudomonadales | Moraxellaceae | *Acinetobacter calcoaceticus* | 9 | Ae |
|  |  |  | *Acinetobacter haemolyticus* | 14 | Ae |
|  |  |  | *Acinetobacter johnsonii* | 14 | Ae |
|  |  |  | *Acinetobacter junii* | 13 | Ae |
|  |  |  | *Acinetobacter lwoffii* | 6 | Ae |
|  |  |  | *Acinetobacter radioresistens* | 1 | Ae |
|  |  |  | *Acinetobacter schindleri* | 17 | Ae |
|  |  |  | *Enhydrobacter aerosaccus* | 2 | Ae |
|  |  |  | *Moraxella catarrhalis* | 9 | Ae |
|  |  |  | *Psychrobacter* | 1 | Ae |
|  |  | Pseudomonadaceae | *Pseudomonas aeruginosa* | 5 | Ae |
|  |  |  | *Pseudomonas fluorescens* | 1 | Ae |
|  |  |  | *Pseudomonas fragi* | 3 | Ae |
|  |  |  | *Pseudomonas geniculata* | 13 | Ae |
|  |  |  | *Pseudomonas mendocina* | 1 | Ae |
|  |  |  | *Pseudomonas putida* | 11 | Ae |
|  |  |  | *Pseudomonas stutzeri* | 1 | Ae |
|  |  |  | *Pseudomonas synxantha* | 6 | Ae |
|  |  |  | *Pseudomonas taiwanensis* | 7 | Ae |
|  | Xanthomonadales | Xanthomonadaceae | *Lysobacter gummosus* | 1 | Ae |
|  |  |  | *Pseudoxanthomonas mexicana* | 2 | Ae |
|  |  |  | *Stenotrophomonas acidaminiphila* | 3 | Ae |
|  |  |  | *Stenotrophomonas maltophilia* | 16 | Ae |
|  |  |  | *Thermomonas fusca* | 1 | Ae |
|  |  |  | *Xanthomonas vesicatoria* | 9 | Ae |
| Mollicutes | Entomoplasmatales | Spiroplasmataceae | *Spiroplasma gent* | 3 | Ae |
| Opitutae | Opitutales | Opitutaceae | *Opitutus terrae* | 1 | An |

**Table S1.** Bacterial species sampled across the 21 cross-sectional samples. Occupancy denotes the number of samples a given species was detected in. Respiration - Ae, denotes aerobe; An, Anerobe^1^. Only strict anaerobes were classified as anaerobes, whereas aerobes, facultative anaerobes, and microaerophiles were classified as aerobes, as described previously (10).
