# Supplementary figures and images for: Ascitic Microbiota Composition Is Correlated with Clinical Severity in Cirrhosis with Portal Hypertension
Source: PLoS One. 2013 Sep 25;8(9):e74884. doi: 10.1371/journal.pone.0074884 (PMC3783492; doi:10.1371/journal.pone.0074884)

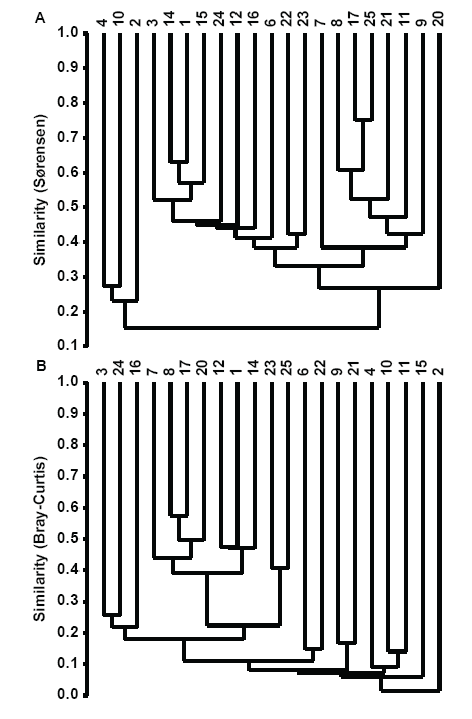

Supplement: Figure S2 — Cluster diagrams of bacterial community composition in the 21 patients. Patient species profiles were compared using the (A) Sørensen and (B) Bray-Curtis quantitative indices of similarity and average linkage clustering. (TIF) [file pone.0074884.s002.tif]
