# Supplementary material for: Ascitic Microbiota Composition Is Correlated with Clinical Severity in Cirrhosis with Portal Hypertension
Source: PLoS One. 2013 Sep 25;8(9):e74884. doi: 10.1371/journal.pone.0074884 (PMC3783492; doi:10.1371/journal.pone.0074884)
Supplement: Methods S1 — Full details of methods used in nucleic acid extraction, quantitative PCR, 16S rRNA gene pyrosequencing, and statistical analysis. (DOC) [file pone.0074884.s004.doc]

**Methods S1**

*DNA extraction protocol*

Nucleic acid extractions were performed on 500 μL cell suspensions. Guanidinium thiocyanate–EDTA–sarkosyl (500 μL) and PBS (500 μL), pH 8.0, were added to samples. Cell disruption was achieved using a Fastprep-24 Instrument (MP Biomedicals Europe, Illkirch, France) 6.5 m/s, 60 s, followed by incubation at 90 °C for 1 min and −20 °C for 5 min. Cell debris was pelleted by centrifugation at 12 000 × *g* for 2 min at 4 °C. Supernatant was transferred to a fresh microfuge tube. NaCl (to a final concentration of 0.5 mol/L and polyethylene glycol (to a final concentration of 15%) were added and DNA precipitated at 4 °C for 30 min. DNA was pelleted by centrifugation at 12 000 × *g* for 2min at 4 °C and resuspended in 300 μL of sterile distilled water. Samples were heated at 90 °C for 30 s and vortexed. Phenol/chloroform (1:1) (300 μL) was added, and samples were vortexed for 20 s before centrifugation at 12 000 × *g* at 4 °C for 3min. The upper phase was then transferred to a fresh microfuge tube. Total DNA was then precipitated by the addition of an equal volume of isopropanol, a 0.1-volume 10 mol/L ammonium acetate, and 1 μL of GenElute linear polyacrylamide (Sigma-Aldrich, Gillingham, UK) and incubated at −20 °C for 25 min. DNA was pelleted by centrifugation at 12 000 × g at 4 °C for 5 min. Pelleted DNA was then washed 3 times in 70% ethanol, dried, and resuspended in 50 μL of sterile distilled water. DNA extracts were quantified using the Picodrop Microlitre Spectrophotometer (GRI, Braintree, UK). Negative controls, consisting of sterile water, were included in the PMA treatment, DNA extraction, and PCR amplification steps.

*Quantitative PCR*

Total bacterial density was determined using a Taqman assay, in which a 466 bp fragment of the 16S ribosomal RNA gene was amplified, as described previously (1), and applied previously in the analysis of ascites (2). Bacterial primers (EubF: 5'-TCCTACGGGAGGCAGCAGT-3', EubR: 5'-GGACTACCAGGGTATCTAATCCTGTT-3') were used at a concentration of 100 nM each, and the probe (EubPr: 5'-FAM-CGTATTACCGCGGCTGCTGGCAC-TAMRA-3') at a concentration of 250 nM. PCR reactions were carried out in a total volume of 25 μl in Taqman® Universal PCR Mastermix (Applied Biosystems, Warrington, UK). Quantitative PCR assays were carried out using the Rotorgene 6000 (Qiagen, Crawley, UK) with a temperature profile of 50 °C for 2 min, 95 °C for 10 min, followed by 45 cycles at 95 °C for 15 s and 60 °C for 60 s. All quantitative (Q)PCR analyses were performed in triplicate, with densities (cfu/ml) determined by comparison with standard curves generated from cultures of *E. coli* (NCTC 12241/ATCC 25922).

*Pyrosequencing*

Bacterial tag-encoded FLX amplicon pyrosequencing (bTEFAP) was performed as described previously using Gray28F 5'-TTTGATCNTGGCTCAG-3' and Gray519r 5'-GTNTTACNGCGGCKGCTG-3'). Initial generation of the sequencing library involved a one-step PCR of 30 cycles, using a mixture of Hot Start and HotStar high fidelity Taq DNA polymerase, as described previously (3). Tag-encoded FLX amplicon pyrosequencing analyses utilized Roche 454 FLX instrument with Titanium reagents, titanium procedures performed at the Research and Testing Laboratory (Lubbock, TX) using RTL protocols (www.researchandtesting.com).

Following sequencing, all failed sequence reads, low quality sequence ends and tags and primers were removed. Sequences with ambiguous base calls, sequences with homopolymers > 6bp were removed. Further, any non-bacterial ribosomal sequences and chimeras using B2C2 (4) as described previously (3). To determine the identity of bacterial species in the remaining sequences, sequences were de-noised, assembled into OUT clusters at 97% identity, and queried using a distributed .N*Et al*gorithm that utilizes Blastn+ (KrakenBLAST www.krakenblast.com) against a database of high quality 16S rRNA gene bacterial sequences. Using a .NET and C# analysis pipeline the resulting BLASTn+ outputs were compiled, data reduction analysis performed, and sequence identity classification carried out, as described previously (3).

Species richness (S*) was estimated as previously described (5). Differences in *S** were computed using the re-sampling randomization method (6) and as previously described (van der Gast, 2011a). As pairwise comparisons will be affected by large differences in sample size (*N*) (7), an Excel macro-program was written to apply Solow’s method to the pairwise comparison of *S** with a uniform sub-sample size (*n* = 1901 sequences). The re-sampling was repeated 1000 times and the mean similarity coefficients and standard deviation were taken. The value of *n* = 1901 was chosen to match to the smallest sample size. Regression analysis, coefficients of determination (*r*^2^), residuals and significance (*P*) were calculated using Minitab software (version 14.20, Minitab, University Park, PA, USA). Boxplots were generated using the XLSTAT (version 2011, Addinsoft, Paris, France) program. The Sørensen presence/absence and Bray-Curtis quantitative indices of similarity and subsequent average linkage clustering of community profiles were performed using the PAST (palaeontological statistics program, version 2.16) software program, available from the University of Oslo website link (http://folk.uio.no/ohammer/past) run by Øyvind Hammer. Similarity of Percentages (SIMPER) analysis was used to determine the contribution of each species to the observed similarity between samples (Bray-Curtis measure). SIMPER analyses were performed as previously described (8) using Community Analysis Package (version 4, Pisces Conservation Ltd., Lymington, UK). Mantel tests were performed as previously described (9).

| **Class** | **Order** | **Family** | **Taxon name** | **Occup.** | **Resp.** |
| --- | --- | --- | --- | --- | --- |
| Acidobacteria | Acidobacteriales | Acidobacteriaceae | *Terriglobus roseus* | 2 | Ae |
| Actinobacteria | Actinomycetales | Actinomycetaceae | *Actinomyces hongkongensis* | 2 | Ae |
|  |  |  | *Actinomyces johnsonii* | 1 | Ae |
|  |  |  | *Actinomyces oris* | 1 | Ae |
|  |  |  | *Actinotalea fermentans* | 1 | Ae |
|  |  | Bifidobacteriaceae | *Bifidobacterium longum* | 2 | An |
|  |  |  | *Bifidobacterium thermacidophilum* | 1 | An |
|  |  | Brevibacteriaceae | *Brevibacterium aureum* | 1 | Ae |
|  |  | Corynebacteriaceae | *Corynebacterium afermentans* | 3 | Ae |
|  |  |  | *Corynebacterium amycolatum* | 1 | Ae |
|  |  |  | *Corynebacterium confusum* | 1 | Ae |
|  |  |  | *Corynebacterium genitalium* | 2 | Ae |
|  |  |  | *Corynebacterium kroppenstedtii* | 1 | Ae |
|  |  |  | *Corynebacterium mucifaciens* | 3 | Ae |
|  |  |  | *Corynebacterium tuberculostearicum* | 4 | Ae |
|  |  |  | *Corynebacterium tuscaniae* | 1 | Ae |
|  |  |  | *Corynebacterium vitaeruminis* | 2 | Ae |
|  |  | Cryptosporangiaceae | *Cryptosporangium japonicum* | 1 | Ae |
|  |  | Geodermatophilaceae | *Blastococcus saxobsidens* | 4 | Ae |
|  |  | Kineosporiaceae | *Kineococcus radiotolerans* | 1 | Ae |
|  |  |  | *Kineosporia mikuniensis* | 1 | Ae |
|  |  | Microbacteriaceae | *Agrococcus jejuensis* | 2 | Ae |
|  |  |  | *Microbacterium barkeri* | 1 | Ae |
|  |  |  | *Microbacterium flavescens* | 1 | Ae |
|  |  |  | *Rathayibacter tritici* | 1 | Ae |
|  |  | Micrococcaceae | *Arthrobacter cumminsii* | 1 | Ae |
|  |  |  | *Arthrobacter nicotinovorans* | 2 | Ae |
|  |  |  | *Kocuria palustris* | 1 | Ae |
|  |  |  | *Micrococcus luteus* | 2 | Ae |
|  |  |  | *Rothia mucilaginosa* | 2 | Ae |
|  |  | Micromonosporaceae | *Micromonospora echinospora* | 1 | Ae |
|  |  |  | *Micromonospora fulviviridis* | 1 | Ae |
|  |  |  | *Virgisporangium ochraceum* | 1 | Ae |
|  |  | Nocardiaceae | *Rhodococcus rhodochrous* | 1 | Ae |
|  |  | Nocardioidaceae | *Aeromicrobium ponti* | 1 | Ae |
|  |  |  | *Marmoricola* | 1 | Ae |
|  |  |  | *Nocardioides oleivorans* | 2 | Ae |
|  |  |  | *Propionibacterium acidifaciens* | 1 | Ae |
|  |  |  | *Propionibacterium acnes* | 20 | Ae |
|  |  |  | *Propionibacterium propionicum* | 2 | Ae |
|  |  | Nocardiopsaceae | *Nocardiopsis composta* | 1 | Ae |
|  |  | Pseudonocardiaceae | *Amycolatopsis halotolerans* | 1 | Ae |
|  |  |  | *Goodfellowiella coeruleoviolacea* | 1 | Ae |
|  |  |  | *Pseudonocardia callicarpae* | 1 | Ae |
|  |  | Sanguibacteraceae | *Sanguibacter inulinus* | 1 | Ae |
|  |  | Streptomycetaceae | *Streptomyces bluensis* | 1 | Ae |
|  |  |  | *Streptomyces laceyi* | 1 | Ae |
|  |  |  | *Streptomyces thermolineatus* | 1 | Ae |
| Actinobacteria | Rubrobacterales | Rubrobacteraceae | *Rubrobacter radiotolerans* | 1 | Ae |
| *Table S1 continued* |  |  |  |  |  |
| **Class** | **Order** | **Family** | **Taxon name** | **Occup.** | **Resp.** |
|  |  |  | *Rubrobacter xylanophilus* | 1 | Ae |
| Bacteroidia | Bacteroidales | Bacteroidaceae | *Bacteroides acidifaciens* | 4 | An |
|  |  |  | *Bacteroides plebeius* | 3 | An |
|  |  |  | *Bacteroides thetaiotaomicron* | 2 | An |
|  |  | Porphyromonadaceae | *Porphyromonas catoniae* | 2 | An |
|  |  |  | *Prevotella enoeca* | 1 | An |
| Flavobacteria | Flavobacteriales | Flavobacteriaceae | *Chryseobacterium daecheongense* | 8 | Ae |
|  |  |  | *Chryseobacterium hominis* | 5 | Ae |
|  |  |  | *Chryseobacterium joostei* | 15 | Ae |
|  |  |  | *Coenonia anatina* | 1 | Ae |
|  |  |  | *Flavobacterium aquatile* | 1 | Ae |
|  |  |  | *Flavobacterium columnare* | 1 | Ae |
|  |  |  | *Flavobacterium succinicans* | 2 | Ae |
|  |  |  | *Maribacter goseongensis* | 1 | Ae |
|  |  |  | *Riemerella anatipestifer* | 15 | Ae |
|  |  |  | *Sejongia* | 1 | Ae |
|  |  |  | *Tenacibaculum japonica* | 1 | Ae |
|  |  |  | *Zhouia amylolytica* | 1 | Ae |
| Sphingobacteria | Sphingobacteriales | Chitinophagaceae | *Chitinophaga arvensicola* | 2 | Ae |
|  |  |  | *Chitinophaga filiformis* | 2 | Ae |
|  |  |  | *Chitinophaga sancti* | 1 | Ae |
|  |  |  | *Segetibacter* | 1 | Ae |
| Sphingobacteria | Sphingobacteriales | Sphingobacteriaceae | *Pedobacter cryoconitis* | 1 | Ae |
|  |  |  | *Pedobacter rhizospharae* | 1 | Ae |
|  |  |  | *Sphingobacterium composta* | 1 | Ae |
| Cyanobacteria | Prochlorophytes | Prochlorococcaceae | *Prochlorococcus* | 1 | Ae |
|  |  |  | *Prochlorococcus marinus* | 1 | Ae |
| Oscillatoriophycideae | Oscillatoriales | Phormidiaceae | *Geitlerinema* | 4 | Ae |
| Bacilli | Bacillales | Alicyclobacillaceae | *Alicyclobacillus pomorum* | 1 | Ae |
|  |  | Bacillaceae | *Bacillus arbutinivorans* | 1 | Ae |
|  |  |  | *Bacillus clausii* | 1 | Ae |
|  |  |  | *Geobacillus stearothermophilus* | 4 | Ae |
|  |  |  | *Geobacillus thermoparaffinivorans* | 2 | Ae |
|  |  | Listeriaceae | *Brochothrix thermosphacta* | 3 | Ae |
|  |  | Staphylococcaceae | *Staphylococcus epidermidis* | 8 | Ae |
|  |  |  | *Staphylococcus hominis* | 2 | Ae |
|  |  | Thermoactinomycetaceae | *Thermoactinomyces vulgaris* | 1 | Ae |
|  | Gemellales | Gemellaceae | *Gemella haemolysans* | 1 | Ae |
|  |  |  | *Gemella sanguinis* | 1 | Ae |
|  | Lactobacillales | Aerococcaceae | *Abiotrophia defectiva* | 2 | Ae |
|  |  | Carnobacteriaceae | *Alloiococcus otitis* | 1 | Ae |
|  |  |  | *Carnobacterium maltaromaticum* | 3 | Ae |
|  |  |  | *Granulicatella adiacens* | 1 | Ae |
|  |  |  | *Granulicatella elegans* | 1 | Ae |
|  |  |  | *Marinilactibacillus psychrotolerans* | 1 | Ae |
|  |  | Enterococcaceae | *Enterococcus cecorum* | 1 | Ae |
|  |  | Lactobacillaceae | *Aerococcus viridans* | 1 | Ae |
|  |  | Leuconostocaceae | *Leuconostoc citreum* | 2 | Ae |
|  |  | Streptococcaceae | *Streptococcus cristatus* | 8 | Ae |
|  |  |  | *Streptococcus mitis* | 10 | Ae |
| *Table S1 continued* |  |  |  |  |  |
| **Class** | **Order** | **Family** | **Taxon name** | **Occup.** | **Resp.** |
|  |  |  | *Streptococcus oralis* | 10 | Ae |
|  |  |  | *Streptococcus pneumoniae* | 10 | Ae |
|  |  |  | *Streptococcus thermophilus* | 1 | Ae |
| Clostridia | Clostridiales | Clostridiaceae | *Anaerococcus hydrogenalis* | 1 | An |
|  |  |  | *Anaerococcus octavius* | 1 | An |
|  |  |  | *Anaerococcus prevotii* | 1 | An |
|  |  |  | *Clostridium bolteae* | 1 | An |
|  |  |  | *Clostridium botulinum* | 1 | An |
|  |  |  | *Clostridium cellobioparum* | 1 | An |
|  |  |  | *Clostridium hveragerdense* | 1 | An |
|  |  |  | *Clostridium kluyveri* | 11 | An |
|  |  |  | *Clostridium straminisolvens* | 2 | An |
|  |  |  | *Finegoldia magna* | 4 | An |
|  |  | Eubacteriaceae | *Acetobacterium carbinolicum* | 3 | An |
|  |  |  | *Eubacterium minutum* | 1 | An |
|  |  |  | *Eubacterium rectale* | 6 | An |
|  |  |  | *Eubacterium sulci* | 1 | An |
|  |  | Heliobacteriaceae | *Heliobacterium modesticaldum* | 1 | An |
|  |  | Lachnospiraceae | *Roseburia faecis* | 2 | An |
|  |  | Ruminococcaceae | *Anaerotruncus colihominis* | 1 | An |
|  |  |  | *Faecalibacterium prausnitzii* | 7 | An |
|  |  |  | *Ruminococcus torques* | 1 | An |
| Erysipelotrichi | Erysipelotrichales | Erysipelotrichaceae | *Allobaculum stercoricanis* | 1 | An |
| Negativicutes | Selenomonadales | Acidaminococcaceae | *Phascolarctobacterium* | 2 | An |
|  |  | Veillonellaceae | *Anaerospora hongkongensis* | 3 | An |
|  |  |  | *Desulfosporomusa polytropa* | 7 | An |
|  |  |  | *Dialister micraerophilus* | 1 | An |
|  |  |  | *Sporomusa ovata* | 1 | An |
|  |  |  | *Veillonella montpellierensis* | 1 | An |
|  |  |  | *Veillonella ratti* | 3 | An |
| Fusobacteria | Fusobacteriales | Fusobacteriaceae | *Fusobacterium nucleatum* | 1 | An |
| Nitrospira | Nitrospirales | Nitrospiraceae | *Nitrospira moscoviensis* | 1 | Ae |
| Planctomycea | Gemmatales | Gemmataceae | *Gemmata obscuriglobus* | 4 | Ae |
| Alphaproteobacteria | Caulobacterales | Caulobacteraceae | *Caulobacter leidyia* | 1 | Ae |
|  |  |  | *Asticcacaulis biprosthecium* | 1 | Ae |
|  |  |  | *Brevundimonas diminuta* | 1 | Ae |
|  | Rhizobiales | Aurantimonadaceae | *Aurantimonas altamirensis* | 1 | Ae |
|  |  | Bradyrhizobiaceae | *Bradyrhizobium elkanii* | 3 | Ae |
|  |  | Brucellaceae | *Ochrobactrum intermedium* | 1 | Ae |
|  |  | Hyphomicrobiaceae | *Devosia ginsengisoli* | 2 | Ae |
|  |  |  | *Devosia riboflavina* | 2 | Ae |
|  |  |  | *Hyphomicrobium* | 2 | Ae |
|  |  |  | *Rhodoplanes* | 3 | Ae |
|  |  | Methylobacteriaceae | *Methylobacterium jeotgali* | 4 | Ae |
|  |  |  | *Methylobacterium variabile* | 1 | Ae |
|  |  | Methylocystaceae | *Methylosinus trichosporium* | 2 | Ae |
|  |  | Phyllobacteriaceae | *Phyllobacterium myrsinacearum* | 2 | Ae |
|  |  | Rhizobiaceae | *Rhizobium sullae* | 1 | Ae |
|  | Rhodobacterales | Rhodobacteraceae | *Paracoccus aminophilus* | 1 | Ae |
|  |  |  | *Paracoccus versutus* | 1 | Ae |
| *Table S1 continued* |  |  |  |  |  |
| **Class** | **Order** | **Family** | **Taxon name** | **Occup.** | **Resp.** |
|  | Rhodospirillales | Acetobacteraceae | *Roseomonas lacus* | 1 | Ae |
|  |  |  | *Roseomonas mucosa* | 1 | Ae |
|  |  |  | *Roseomonas terrae* | 1 | Ae |
|  | Rickettsiales | Anaplasmataceae | *Wolbachia pipientis* | 1 | Ae |
|  | Sphingomonadales | Erythrobacteraceae | *Porphyrobacter tepidarius* | 1 | Ae |
|  |  |  | *Kaistobacter terrae* | 2 | Ae |
|  |  |  | *Novosphingobium* | 11 | Ae |
|  |  |  | *Sphingobium fuliginis* | 3 | Ae |
|  |  |  | *Sphingobium yanoikuyae* | 6 | Ae |
|  |  |  | *Sphingomonas aerolata* | 1 | Ae |
|  |  |  | *Sphingomonas melonis* | 1 | Ae |
|  |  |  | *Sphingomonas yabuuchiae* | 1 | Ae |
| Betaproteobacteria | Burkholderiales | Alcaligenaceae | *Achromobacter xylosoxidans* | 3 | Ae |
|  |  | Burkholderiaceae | *Cupriavidus campinensis* | 1 | Ae |
|  |  |  | *Cupriavidus necator* | 3 | Ae |
|  |  |  | *Ralstonia pickettii* | 15 | Ae |
|  |  |  | *Roseateles depolymerans* | 7 | Ae |
|  |  | Comamonadaceae | *Acidovorax avenae* | 2 | Ae |
|  |  |  | *Acidovorax citrulli* | 4 | Ae |
|  |  |  | *Acidovorax delafieldii* | 2 | Ae |
|  |  |  | *Acidovorax facilis* | 19 | Ae |
|  |  |  | *Acidovorax temperans* | 14 | Ae |
|  |  |  | *Comamonas aquatica* | 2 | Ae |
|  |  |  | *Comamonas denitrificans* | 6 | Ae |
|  |  |  | *Comamonas testosteroni* | 13 | Ae |
|  |  |  | *Delftia* | 5 | Ae |
|  |  |  | *Hydrogenophaga palleronii* | 1 | Ae |
|  |  |  | *Pelomonas* | 2 | Ae |
|  |  |  | *Pelomonas puraquae* | 3 | Ae |
|  |  |  | *Schlegelella thermodepolymerans* | 2 | Ae |
|  |  |  | *Variovorax paradoxus* | 1 | Ae |
|  |  | Oxalobacteraceae | *Massilia timonae* | 4 | Ae |
|  | Methylophilales | Methylophilaceae | *Methylobacillus aminovorus* | 1 | Ae |
|  |  |  | *Methylobacillus flagellatus* | 4 | Ae |
|  | Neisseriales | Neisseriaceae | *Neisseria sicca* | 2 | Ae |
|  |  |  | *Neisseria subflava* | 4 | Ae |
|  | Rhodocyclales | Rhodocyclaceae | *Azonexus fungiphilus* | 1 | Ae |
|  |  |  | *Zoogloea ramigera* | 1 | Ae |
| Deltaproteobacteria | Desulfobacterales | Desulfobacteraceae | *Desulfatibacillum alkenivorans* | 1 | Ae |
|  | Desulfobacterales | Desulfovibrionaceae | *Desulfovibrio burkinensis* | 1 | Ae |
|  |  |  | *Desulfovibrio desulfuricans* | 4 | Ae |
|  | Myxococcales | Nannocystaceae | *Nannocystis exedens* | 1 | Ae |
|  |  | Polyangiaceae | *Chondromyces crocatus* | 1 | Ae |
| Gammaproteobacteria | Aeromonadales | Aeromonadaceae | *Aeromonas hydrophila* | 1 | Ae |
|  |  |  | *Aeromonas punctata* | 10 | Ae |
|  |  |  | *Aeromonas salmonicida* | 1 | Ae |
|  |  |  | *Aeromonas veronii* | 2 | Ae |
|  | Alteromonadales | Shewanellaceae | *Shewanella putrefaciens* | 4 | Ae |
|  | Chromatiales | Chromatiaceae | *Rheinheimera aquimaris* | 1 | Ae |
|  |  | Ectothiorhodospiraceae | *Thioalkalivibrio denitrificans* | 2 | Ae |
| *Table S1 continued* |  |  |  |  |  |
| **Class** | **Order** | **Family** | **Taxon name** | **Occup.** | **Resp.** |
|  | Enterobacteriales | Enterobacteriaceae | *Aquamonas fontana* | 1 | Ae |
|  |  |  | *Citrobacter farmeri* | 1 | Ae |
|  |  |  | *Erwinia persicina* | 1 | Ae |
|  |  |  | *Escherichia coli* | 4 | Ae |
|  |  |  | *Klebsiella oxytoca* | 5 | Ae |
|  |  |  | *Proteus mirabilis* | 1 | Ae |
|  |  |  | *Yersinia mollaretii* | 2 | Ae |
|  | Methylococcales | Crenotrichaceae | *Crenothrix polyspora* | 1 | Ae |
|  | Pasteurellales | Pasteurellaceae | *Aggregatibacter segnis* | 1 | Ae |
|  |  |  | *Haemophilus parainfluenzae* | 1 | Ae |
|  | Pseudomonadales | Moraxellaceae | *Acinetobacter calcoaceticus* | 9 | Ae |
|  |  |  | *Acinetobacter haemolyticus* | 14 | Ae |
|  |  |  | *Acinetobacter johnsonii* | 14 | Ae |
|  |  |  | *Acinetobacter junii* | 13 | Ae |
|  |  |  | *Acinetobacter lwoffii* | 6 | Ae |
|  |  |  | *Acinetobacter radioresistens* | 1 | Ae |
|  |  |  | *Acinetobacter schindleri* | 17 | Ae |
|  |  |  | *Enhydrobacter aerosaccus* | 2 | Ae |
|  |  |  | *Moraxella catarrhalis* | 9 | Ae |
|  |  |  | *Psychrobacter* | 1 | Ae |
|  |  | Pseudomonadaceae | *Pseudomonas aeruginosa* | 5 | Ae |
|  |  |  | *Pseudomonas fluorescens* | 1 | Ae |
|  |  |  | *Pseudomonas fragi* | 3 | Ae |
|  |  |  | *Pseudomonas geniculata* | 13 | Ae |
|  |  |  | *Pseudomonas mendocina* | 1 | Ae |
|  |  |  | *Pseudomonas putida* | 11 | Ae |
|  |  |  | *Pseudomonas stutzeri* | 1 | Ae |
|  |  |  | *Pseudomonas synxantha* | 6 | Ae |
|  |  |  | *Pseudomonas taiwanensis* | 7 | Ae |
|  | Xanthomonadales | Xanthomonadaceae | *Lysobacter gummosus* | 1 | Ae |
|  |  |  | *Pseudoxanthomonas mexicana* | 2 | Ae |
|  |  |  | *Stenotrophomonas acidaminiphila* | 3 | Ae |
|  |  |  | *Stenotrophomonas maltophilia* | 16 | Ae |
|  |  |  | *Thermomonas fusca* | 1 | Ae |
|  |  |  | *Xanthomonas vesicatoria* | 9 | Ae |
| Mollicutes | Entomoplasmatales | Spiroplasmataceae | *Spiroplasma gent* | 3 | Ae |
| Opitutae | Opitutales | Opitutaceae | *Opitutus terrae* | 1 | An |

**Table S1.** Bacterial species sampled across the 21 cross-sectional samples. Occupancy denotes the number of samples a given species was detected in. Respiration - Ae, denotes aerobe; An, Anerobe^1^.

^1^ Only strict anaerobes were classified as anaerobes, whereas aerobes, facultative anaerobes, and microaerophiles were classified as aerobes, as described previously (10).

**Figure S1.** Ascites fluid was passed through a 20 μm filter to remove any bacterial cells. To three 2ml portions of filtered ascites were added one of the following; 1 x 10^6^ viable log-phase *Escherichia coli* (NCTC 12241/ATCC 25922)*,* 1 x 10^6^ viable log-phase *Staphylococcus. aureus* (MSSA: ATCC 29213)*,* or 1 x 10^6^ viable log-phase *Acinetobacter baumanii* (NCTC 12156)*.* Cell suspensions were divided in two, and bacterial cells heat-killed by incubation at 90ºC, 20 min. For each bacterial species, one portion was treated with PMA, as described for clinical samples, followed by DNA extraction from all cell suspension. PCR amplification of a ~919 bp region of the 16S rRNA gene was performed as described previously (11), with amplicons visualised by gel electrophoresis (above). Lane 1 – no template negative control. Lanes 2, 4, and 6 – amplification reactions using DNA extracted from heat-killed *A. baumanii*, *S. aureus*, and *E. coli*, respectively, as template. Lanes 3, 5, and 7 - amplification reactions using DNA extracted from PMA treated heat-killed *A. baumanii*, *S. aureus*, and *E. coli*, respectively.

**Figure S2.** Cluster diagrams of bacterial community composition in the 21 patients. Patient species profiles were compared using the (A) Sørensen and (B) Bray-Curtis quantitative indices of similarity and average linkage clustering.

**References**

1. Nadkarni MA, Martin FE, Jacques NA, Hunter N. Determination of bacterial load by real-time PCR using a broad-range (universal) probe and primers set. Microbiol 2002;148:257-266.
2. Rogers GB, Marsh P, Stressmann AF, Allen CE, Daniels TVW, Carroll MP, Bruce KD. The exclusion of dead bacterial cells is essential for accurate molecular analysis of clinical samples. Eur J Clin Microbiol Infect Dis 2010;16:1656-8.
3. Dowd SE, Wolcott RD, Sun Y, McKeehan T, Smith E, Rhoads D. Polymicrobial nature of chronic diabetic foot ulcer biofilm infections determined using bacterial tag encoded FLX amplicon pyrosequencing (bTEFAP). *PLOS One* 2008;3:e3326.
4. Gontcharova V, Youn E, Sun Y, Wolcott RD, Dowd SE. A comparison of bacterial composition in diabetic ulcers and contralateral intact skin. *Open Microbiol J* 2010;4**:** 8-19.
5. Edwards ML, Lilley AK, Timms-Wilson TH, Thompson IP, Cooper I. Characterisation of the culturable heterotrophic bacterial community in a small eutrophic lake (Priest Pot). FEMS Microbiol Ecol *2001;*35: 295-304.
6. Solow AR. A simple test for change in community structure. J Anim Ecol 1993;62: 191-193.
7. Gihring TM, Green SJ, Schadt CW. Massively parallel rRNA gene sequencing exacerbates the potential for biased community diversity comparisons due to variable library sizes. Environ Microbiol 2012;14: 285-290.
8. Clarke, K. R. Non-parametric multivariate analyses of changes in community structure. Austral J Ecol 1993;199318:117–143.
9. van der Gast CJ, Gosling P, Tiwari B, Bending GD. Spatial scaling of arbuscular mycorrhizal fungal diversity is affected by farming practice. Environ Microbiol 2011;13:241-9.
10. van der Gast CJ, Walker AW, Stressmann FA, Rogers GB, Daniels TWV, Carroll MP, Parkhill J, Bruce KD. Partitioning core and satellite taxa from within cystic fibrosis lung bacterial communities. 2010, *ISME* May;5(5):780-91.
11. Rogers GB, Stressmann FA, Koller G, Daniels T, Carroll MP, Bruce KD. Assessing the diagnostic importance of nonviable bacterial cells in respiratory infections. *Diagnostic Microbiology and Infectious Disease* 2008, 62(2): 133-141.
